# Three YXXL Sequences of a Bovine Leukemia Virus Transmembrane Protein are Independently Required for Fusion Activity by Controlling Expression on the Cell Membrane

**DOI:** 10.3390/v11121140

**Published:** 2019-12-10

**Authors:** Ryosuke Matsuura, Kazunori Inabe, Hiroyuki Otsuki, Kazuo Kurokawa, Naoshi Dohmae, Yoko Aida

**Affiliations:** 1 Viral Infectious Diseases Unit, RIKEN, 2-1 Hirosawa, Wako, Saitama 351-0198, Japan; 2 Biomolecular Characterization Unit, RIKEN Center for Sustainable Resource Science, 2-1 Hirosawa, Wako, Saitama 351-0198, Japan; 3 Laboratory of Viral Infectious Diseases, Department of Computational Biology and Medical Sciences, Graduate School of Frontier Sciences, The University of Tokyo, 2-1 Hirosawa, Wako, Saitama 351-0198, Japan; 4 Live Cell Super-Resolution Imaging Research Team, RIKEN Center for Advanced Photonics, 2-1 Hirosawa, Wako, Saitama 351-0198, Japan; 5 Nakamura Laboratory, Baton Zone program, Riken Cluster for Science, Technology and Innovation Hub, 2-1 Hirosawa, Wako, Saitama 351-0198, Japan

**Keywords:** BLV, YXXL sequence, syncytia formation, Env distribution, virion incorporation, internalization, endosome, membrane binding

## Abstract

Bovine leukemia virus (BLV), which is closely related to human T-cell leukemia viruses, is the causative agent of enzootic bovine leukosis, the most common neoplastic disease of cattle. The transmembrane subunit of the BLV envelope glycoprotein, gp30, contains three completely conserved YXXL sequences that fit an endocytic sorting motif. The two N-terminal YXXL sequences are reportedly critical for viral infection. However, their actual function in the viral life cycle remains undetermined. Here, we identified the novel roles of each YXXL sequence. Syncytia formation ability was upregulated by a single mutation of the tyrosine (Tyr) residue in any of the three YXXL sequences, indicating that each YXXL sequence is independently able to regulate the fusion event. The alteration resulted from significantly high expression of gp51 on the cell surface, thereby decreasing the amount of gp51 in early endosomes and further revealing that the three YXXL sequences are independently required for internalization of the envelope (Env) protein, following transport to the cell surface. Moreover, the 2nd and 3rd YXXL sequences contributed to Env protein incorporation into the virion by functionally distinct mechanisms. Our findings provide new insights regarding the three YXXL sequences toward the BLV viral life cycle and for developing new anti-BLV drugs.

## 1. Introduction

The immunoreceptor tyrosine-based activation motif (ITAM) is present in the cytoplasmic tails of several protein components of antigen receptors on T and B cells in addition to the Fc receptor for immunoglobulin E [1]. This motif is denoted as Yxx(L/I)–x6–8–Yxx(L/I), where x corresponds to a variable residue. Several viruses, such as bovine leukemia virus (BLV), which can cause B cell lymphomas or leukemia in cattle, Epstein-Barr virus, which can cause Burkitt’s B cell lymphomas in humans, and human herpesvirus 8, which can cause sarcomas and primary effusion B cell lymphomas in humans, contain ITAMs in their viral proteins [1,2,3]. Notably, these viruses can infect B lymphocytes, as well as non-hematopoietic cells, such as epithelial and endothelial cells, and the ITAM-bearing viral proteins are implicated as candidates for effecting the cellular transformation and cancer induced by these viruses [4]. 

The envelope glycoprotein (Env) of BLV, which constitutes the causative agent of enzootic bovine leukosis, the most common neoplastic disease of cattle, and is closely related to human T-cell leukemia viruses (HTLVs), contains two overlapping copies of the (YXXL/I)_2_ sequence serving as the ITAM at the C-terminal domain of the transmembrane (TM) protein [1,5]. The Env protein of BLV is synthesized as a Pr72 precursor peptide that is glycosylated in the rough-surfaced endoplasmic reticulum and Golgi apparatus [6], and cleaved into two mature proteins, the surface subunit gp51 and the TM subunit gp30 by cellular protease [7]. The gp51 and gp30 proteins form a stable complex through disulfide bonds [8], and are incorporated into budding viral particles [9]. gp51 binds to cationic amino acid transporter 1 (CAT1)/SLC7A1, which acts as a cellular receptor for BLV, and is responsible for its broad host range [10]. In comparison, gp30 contains three distinct domains: An extracellular domain that interacts with gp51 and also contains a stretch of about 12 hydrophobic amino acids at its N-terminus, which is designated as the fusion peptide [11]; a membrane-spanning domain that anchors the gp51–gp30 complex in the plasma membrane of infected cells and the viral membrane [8]; and the cytoplasmic tail, comprised of 58 amino acids and containing the three YXXL sequences that were originally identified as two sets of ITAMs [1]. Studies of chimeric proteins generated by replacing the cytoplasmic tail of CD8-α with that of BLV gp30, and site-directed mutagenesis has suggested that the ITAM in the BLV gp30 cytoplasmic tail is capable of triggering both calcium responses and cytokine production when cross-linked with an antibody to CD8-α, indicating that an ITAM can elicit early and late cellular activation events similar to those normally observed upon antigen-receptor triggering [12]. Moreover, mRNA expression of the spleen tyrosine kinase (Syk), which is directly responsible for ITAM-mediated signaling, was significantly increased in samples from BLV-infected cattle with persistent lymphocytosis induced by BLV, whereas it was decreased in samples from BLV-infected cattle with lymphoma, suggesting that the dynamics of *Syk* mRNA expression is closely related to the progression of BLV-induced disease [13]. However, previous research has indicated that gp30 is not phosphorylated on tyrosine residues in vivo and in vitro [14].

The three YXXL sequences in the BLV gp30 cytoplasmic tail also fit the tyrosine-based motif, YXXφ, wherein x corresponds to a variable residue, and φ is an amino acid with a bulky hydrophobic side chain [15]. The YXXφ motif functions as an endocytic sorting motif and directly binds to the µ2 subunit of adaptor protein-2 (AP2) [16]. This AP2 complex has an essential role in the initiation of clathrin-mediated endocytosis [17]. The Env protein of most retroviruses such as human immunodeficiency virus (HIV), simian immunodeficiency virus (SIV), and HTLV-1 contains only a single YXXφ motif [18,19,20]. In the case of HIV, the YSPL sequence contained in the Env protein is important for viral endocytosis and required for viral replication and infectivity [21].

In comparison, although several studies reported that the YXXL sequences of BLV gp30 are associated with endocytosis of the Env protein [22,23], in vivo, the YXXL sequences of gp30 mediated high proviral loads in experimentally infected sheep [24]. In addition, it has been revealed that mutation of the tyrosine at position 498 to alanine within the 2nd YXXL sequence markedly reduces viral infectivity as a result of decreases in both viral entry and incorporation of the viral envelope protein into virions [15]. Thus, the two N-terminal YXXL sequences among the three YXXL sequences in gp30 appear to play a critical role in viral infection, although their actual function in the viral life cycle has not yet been identified.

However, although the two N-terminal YXXL sequences are essential for signal transduction [12] along with viral infection in cultured cells [15] and experimentally infected sheep [24], the third sequence is not necessary for these activities. Therefore, in the present study, we focused on all three YXXL sequences in their capacity as a tyrosine-based motif, YXXφ, rather than the (YXXL/I)_2_ signaling motif, ITAM. Firstly, we analyzed the role of the three YXXL sequences in syncytia formation, which is an indispensable event in the viral life cycle, and demonstrated that the syncytia formation ability was regulated independently by each tyrosine residue of the 1st, 2nd, and 3rd YXXL sequences. Next, we demonstrated that the alteration of syncytia formation ability resulted from a distribution change of the gp51 protein consequent to a mutation in the tyrosine residue of any of the 1st, 2nd, and 3rd YXXL sequences. Finally, we clarified the effects of the 2nd and 3rd YXXL sequences with regard to the incorporation of the gp51 protein into virions.

## 2. Materials and Methods

### 2.1. Plasmids and Construction

**Figure 1:**
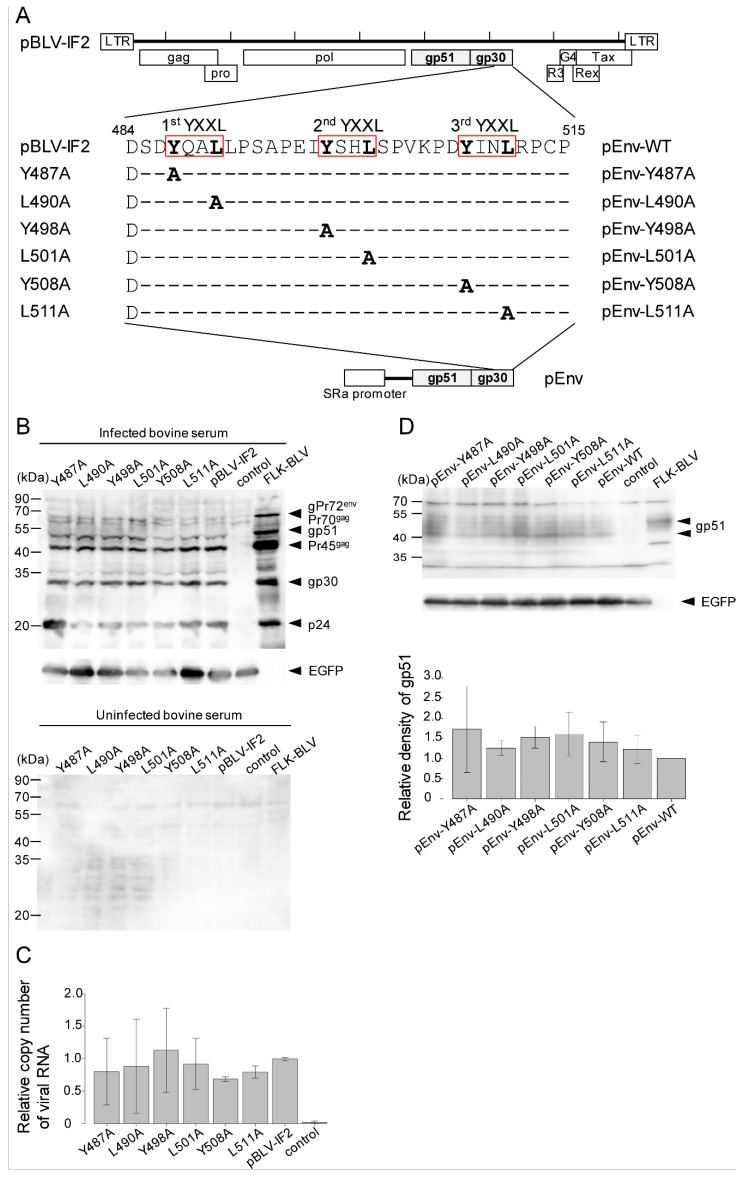
Relevant sequences of bovine leukemia virus (BLV) mutants in an infectious molecular clone, pBLV-IF2, and Envelope glycoprotein (Env) expression plasmid, pEnv, and biological features of mutant forms of BLV following transient transfection of COS-1 cells with both plasmids. (**A**) The amino acid sequence of the cytoplasmic tail of wild-type gp30 is shown at the top; the locations of the three YXXL sequences are indicated. The cytoplasmic tail contains three repeats of the YXXL sequence, denoted as 1st, 2nd, and 3rd. The positions of substitutions by alanine of leucine and tyrosine residues described in this study are indicated under the wild-type sequence. Amino acids identical to those in the latter sequence are indicated by dashes. (**B**,**C**) COS-1 cells (5.0 × 10^5^) were seeded in a 60 mm dish the day prior to transfection and were transfected with either 7.6 µg of wild-type pBLV-IF2, 7.6 µg of mutant pBLV-IF2, or 7.6 µg of the control pBluescript II SK (−) vector together with 0.4 µg of pEGFP-N1 using 32 μL of FuGENE HD. At 48 h after transfection, cells were harvested for western blotting analysis (**B**), and supernatants were collected for quantitative reverse transcription-polymerase chain reaction (RT-qPCR) assay (**C**). A portion of the harvested cells was used for identification of the ratio of green fluorescent protein (GFP)-expressing cells to determine the transfection efficiency, and the remainder were lysed. Lysates with equal numbers of GFP-expressing cells were subjected to western blotting analysis using BLV-infected bovine serum or anti-GFP monoclonal antibody (MAb) (upper panel) or uninfected bovine serum (lower panel) (**B**). The lysate of FLK-BLV cells, which are persistently infected with BLV, was used as a positive control. Positions of the molecular mass marker of the BLV structural protein and EGFP are indicated. Supernatants were used to collect viral RNAs, which were then reverse transcribed, and the copy number of viral RNA was determined by using BLV-CoCoMo-qPCR (**C**). The data shows the relative copy number of viral RNA, which was calculated as the actual copy number/relative ratio of GFP-expressing cells, as indicated in (**B**). Each column and error bar represents the mean ± SD of results for three independent experiments. All values were analyzed by two-way analysis of variance (ANOVA) with Dunnett’s test. (**D**) Cos-1 cells (5.0 × 10^5^) were seeded in a 60 mm dish the day prior to transfection and transfected with either 3.8 µg of pEnv-wild-type (WT), 3.8 µg of pEnv-mutant, or 3.8 µg of the control pME-18neo together with 0.2 µg of pEGFP-N1 using 16 μL of FuGENE HD. At 48 h following transfection, cells were harvested, and a portion as used to identify the ratio of GFP-expressing cells to determine the transfection efficiency, and the remainder were lysed. Lysates with equal numbers of GFP-expressing cells were subjected to western blotting analysis using anti-BLV gp51MAb or anti-GFP MAb followed by horseradish peroxidase-conjugated goat anti-mouse IgG. Positions of the molecular mass marker and of gp51 and EGFP are indicated. For quantification, densities of bands were analyzed using AlphaEaseFC^TM^ software. Densities of gp51 were normalized with those of EGFP. Each column and error bar represents the mean ± SD of density for three independent experiments. All values were analyzed by two-way ANOVA with Dunnett’s test.

The modified version of the infectious molecular clone of BLV, pBLV-IF2, was modified from the original pBLV-IF [25] to be suitable for amplification in *Escherichia coli*. To generate the mutant infectious molecular clones designated Y487A, L490A, Y498A, L501A, Y508A, and L511A, alanine mutations were introduced into the tyrosine or leucine residue of the three YXXL sequences in pBLV-IF2 by site-directed mutagenesis, as shown in Figure 1. In brief, the 3′-half of pBLV-IF2, which contains the gp30 sequence, was subcloned into the *Hind*III and *Kpn*I restriction enzyme sites of a pBluescript II SK (−) vector to yield 3′-BLV/SK. A tyrosine or leucine residue was changed to alanine by polymerase chain reaction (PCR) amplification with 3′-BLV/SK as a template using PrimeSTAR MAX (TaKaRa Bio, Otsu, Japan) and the following primers: Y487Afor, 5′-tctgatGCtcaggccttgctaccat-3′ and Y487Arev, 5′-ggcctgaGCatcagaatcgggttta-3′, for Y487A; L490Afor, 5′-aggccGCTctaccatctgcaccaga-3′ and L490Arev, 5′-gatggtagAGCggcctgataatcagaa-3′ for L490A; Y498Afor, 5′-agagatcGCTtctcacctctccccc-3′ and Y498Arev, 5′-ggtgagaAGCgatctctggtgcagatg-3′, for Y498A; L501Afor, 5′-tctcacGCTtcccccgtcaaacccg-3′ and L501Arev, 5′-cgggggaAGCgtgagagtagatctctg-3′, for L501A; Y508Afor, 5′-cccgatGCTatcaacctccgaccct-3′ and Y508Arev, 5′-ggttgatAGCatcgggtttgacgggg-3′, for Y508A; and L511Afor, 5′-catcaacGCTcgaccctgcccttgat-3′ and L511Arev, 5′-gggtcgAGCgttgatgtaatcgggt-3′, for L511A. The sequences corresponding to the alanine substitution are underlined. The *Hind*III–*Eco*RI fragments, which contain the mutated gp30 of 3′-BLV/SK, were replaced with the corresponding region of pBLV-IF2.

To obtain wild-type (WT) and mutated Env protein expression plasmids, termed pEnv-WT, pEnv-Y487A, pEnv-L490A, pEnv-Y498A, pEnv-L501A, pEnv-Y508A, or pEnv-L511A, the *Xho*I–*Xba*I fragments, which contained the BLV Env sequence of WT and six mutant pBLV-IF2s, were inserted at the *Xho*I and *Xba*I sites of the pME-18 neo vector. pEGFP-N1, which encodes a red-shifted variant of WT green fluorescent protein (GFP) [26], and Flag-mRFP [27] were used for transfection efficiency detection.

### 2.2. Cell Culture and Transfections

COS-1 African green monkey kidney cells(RIKEN BRC: RCB0143), the persistently BLV-infected FLK-BLV cells(Kindly provided Prof. Onuma, M.), HeLa human cervical cancer cells(Riken BRC: RCB0007), CC81 mouse sarcoma virus-transformed cat cells(Kindly provided Prof. Onuma, M.), and the BLV reporter CC81 transfectants CC81-GREMG cells [28] were grown in Dulbecco’s modified Eagle’s medium (Thermo Fisher Scientific, Waltham, MA, USA) supplemented with 10% heat-inactivated fetal bovine serum (Sigma-Aldrich, St. Louis, MO, USA), penicillin, and streptomycin.

For western blotting analysis, quantitative reverse transcription (RT-q)PCR assay, or virus particle concentration assessment, COS-1 cells were transfected with each pBLV-IF2, each pEnv, or control vector together with pEGNP-N1 using FuGENE HD (Promega, Madison, WI, USA). For immunofluorescence microscopy, HeLa cells were transfected with each pBLV-IF2, each pEnv, or control vector using FuGENE HD (Promega). For assaying the formation of syncytia of infectious molecular clones, CC81-GREMG cells were transfected with each pBLV-IF2 or control vector together with Flag-mRFP using 25 kDa linear polyethylenimine (Polysciences, Eppenheim, Germany). For assay of formation of syncytia of the Env protein, CC81 cells were transfected with each pEnv or control vector together with pEGFP-N1 using mixes of Lipofectamine 3000 Transfection Reagent (Thermo Fisher Scientific) and P3000 Enhancer Reagent (Thermo Fisher Scientific).

### 2.3. The Concentration of Virus Particles

Virus particles were collected from the supernatants of COS-1 cells that were transfected with each pBLV-IF2 by centrifugation at 194,000× *g* for 45 min at 4 °C.

### 2.4. Western Blotting Analysis

Transfected COS-1 cells were harvested at 48 h post-transfection, and a fraction was used to determine the ratio of GFP-expressing cells via FACSCalibur™ flow cytometer (BD Japan, Tokyo, Japan). The remainder were lysed, and lysates with equal numbers of GFP-expressing cells were subjected to western blotting analysis as described previously [29]. Collected virus particles were also subjected to western blotting analysis. Subsequently, proteins were transferred to a polyvinylidene difluoride membrane filter (Immobilon; Merck Millipore, Burlington, MA, USA) and incubated with sera from BLV-infected cattle and BLV-uninfected cattle, anti-BLV gp51 monoclonal antibody (MAb) (BLV-2; VMRD, Pullman, WA, USA), anti-BLV p24 MAb (BLV-3; VMRD), or anti GFP MAb (1E4; MBL, Nagoya, Japan). After washing, the membranes were incubated with horseradish peroxidase (HRP)-conjugated goat anti-bovine IgG (Jackson ImmunoResearch Laboratories, Inc., West Grove, PA, USA) or HRP-conjugated goat anti-mouse IgG (Jackson ImmunoResearch Laboratories, Inc.). Densities of bands were analyzed using AlphaEaseFC^TM^ software (Alpha Innotech, San Leandro, CA, USA) and ImageJ software (National Institutes of Health, Bethesda, MD, USA).

### 2.5. RT-qPCR

Viral RNA was isolated from the supernatants of COS-1 cells that were transfected with each pBLV-IF2 or the control pBluescript II SK (−) using the QIAamp Viral RNA Mini Kit (Qiagen, Hilden, Germany) and DNA was removed using the TURBO DNA-free^TM^ Kit (Thermo Fisher Scientific). Viral RNA was reverse transcribed using the High Capacity RNA-to-cDNA Kit (Applied Biosystems, Foster City, CA, USA). Copy number of viral RNA was determined by BLV-CoCoMo-qPCR (RIKEN GENESIS Co., Ltd., Tokyo, Japan) [30,31,32].

### 2.6. Syncytia Formation Assay

The luminescence syncytium induction assay (LuSIA) using CC81-GREMG cells was performed as described previously [28]. CC81-GREMG cells in one well were harvested 20 h post-transfection with pBLV-IF2, and the ratios of RFP-expressing cells were determined via FACSCalibur™ flow cytometer. Two days post-transfection, CC81-GREMG cells in the remaining well were fixed using 3.6% formaldehyde/phosphate-buffered saline (PBS) with 10 µg/mL Hoechst 33342. Fluorescent syncytia expressing enhanced green fluorescent protein (EGFP) were observed using EVOS2 fluorescence microscopy (Thermo Fisher Scientific) and counted using HCS Studio Cell Analysis software (Thermo Fisher Scientific). The fluorescence area of syncytia with EGFP was also measured using HCS Studio Cell Analysis software. To observe fluorescent syncytium at high resolution, CC81-GREMG cells on coverslips were fixed with 3.6% formaldehyde/PBS with 10 µg/mL Hoechst 33342 two days post-transfection and mounted with 90% glycerol/PBS. Fluorescent syncytia were observed using an FV1000 confocal laser scanning microscope (Olympus, Tokyo, Japan).

Conventional syncytia formation assay was performed as described previously [33]. Transfected CC81 cells were fixed using May–Grunwald solution (Merck, Darmstadt, Germany) for 2 min and then stained with May–Grunwald solution diluted with phosphate buffer for 3 min and with diluted Giemsa solution (Sigma-Aldrich) for 15 min. The stained syncytia were counted using a microscope.

### 2.7. Fluorescence Microscopy

To detect cell surface gp51, cells growing on a coverslip in a 12 well plate were treated with 4% paraformaldehyde/PBS for 10 min. To detect intracellular gp51, cells were fixed and permeabilized with 0.5% Triton X-100 for 5 min. Then, fixed cells were incubated with anti-BLV gp51 MAb (BLV-1; VMRD) at room temperature for 1 h. After washing, cells were treated with Alexa Fluor 488-conjugated Goat anti-Mouse IgG (Thermo Fisher Scientific) for 30 min and then stained with Hoechst 33342 (Thermo Fisher Scientific). Samples were mounted with 90% glycerol/PBS and examined on a focal plane near the center of each nucleus using an FV1000 confocal laser scanning microscope. Fluorescence level was analyzed using FV10-ASW 4.02 microscopy software (Olympus). For Z-stack analysis, three images were recorded at 2.0 µm intervals to demonstrate membrane domain-specific localizations of gp51.

To detect localization of gp51 in the early endosome and trans-Golgi network, fixed and permeabilized cells were incubated in a mixture of anti-BLV gp51 MAb (BLV-1; VMRD) with anti-EEA1 polyclonal antibody (PAb) (ab2900; Abcam, Cambridge, UK) or anti-TGN46 PAb (ab50595; Abcam), followed by Alexa Fluor 594-conjugated goat anti-mouse (Thermo Fisher Scientific) and Alexa Fluor 488-conjugated goat anti-rabbit IgG (Thermo Fisher Scientific). Samples were mounted with 90% glycerol/PBS and examined on a focal plane containing the early endosome or trans-Golgi network using an FV1000 confocal laser scanning microscope. The colocalization index on a focal plane containing the early endosome or trans-Golgi network was calculated using FV10-ASW 4.02 microscopy software.

### 2.8. Quantification of Intensity of Env Protein on the Cell Membrane

Fluorescence intensity maps of Env protein in HeLa cells that were transfected with each pBLV-IF2 or pEnv expression plasmid were plotted by line scan measurements through each cell using FV10-ASW 4.02 microscopy software on a focal plane near the center of each nucleus, and peak membrane intensity was determined from these data, as shown in Supplementary Figure S1. The intensity of each cell was normalized by the mean intensity of WT for each experiment. Data were obtained for at least 50 cells.

### 2.9. Quantification of Colocalization

Quantification of Env protein colocalization with EEA1 and TGN46 was performed using Villalta’s algorithm [34]. This algorithm runs using FV10-ASW 4.02 microscopy software on a focal plane containing the early endosome or trans-Golgi network. Data were obtained for at least 50 cells.

### 2.10. Statistical Analysis

Two-way analysis of variance (ANOVA) with Dunnett’s test comparing all samples to WT was used for statistical determinations. *p* values of 0.05 were considered statistically significant.

## 3. Results

### 3.1. Mutations in YXXL Sequences in pBLV-IF2 and Env Expression Plasmids Do not Affect the Expression of Viral Protein and the Release of Virus

To demonstrate the functional role of all of three copies of YXXL sequences on the life cycle of BLV, designated as the 1st, 2nd, and 3rd YXXL sequences, each tyrosine and leucine component of the three YXXL sequences in an infectious molecular clone of BLV, pBLV-IF2, was separately changed to alanine by site-directed mutagenesis (Figure 1A).

To examine the expression of cell-associated viral proteins and the production of viral particles by the YXXL mutant proviruses, COS-1 cells were transiently transfected with either WT or mutant infectious molecular clone Y487A, L490A, Y498A, L501A, Y508A, or L511A. After two days following transfection, we performed western blotting and RT-qPCR analyses. Cell-associated viral proteins, such as p24, gp30, and gp51, were specifically detected by western blotting analysis with serum from BLV-infected cattle (Figure 1B). The expression levels and molecular masses of these mutant viral proteins were equivalent to those of WT detected in analyses of COS-1 cells that had been transfected with WT proviral DNA, which served as positive controls (Figure 1B). In contrast, no specific bands were detected with serum from uninfected cattle. Next, to confirm whether the virus particles were released into the medium of COS-1 cells that had been transfected with either mutant proviral DNAs or WT DNA, viral RNA was detected by RT-qPCR (Figure 1C). The amount of viral RNA did not differ between WT and mutant viruses. These results suggested that none of the six YXXL mutants affected the expression of viral proteins or the formation of viral particles.

To confirm the function of the Env protein in the absence of other viral proteins, WT and mutant Env expression plasmids, termed pEnv-WT, pEnv-Y487A, pEnv-L490A, pEnv-Y498A, pEnv-L501A, pEnv-Y508A, or pEnv-L511A, were constructed by subcloning the WT or YXXL-mutated Env gene from pBLV-IF2, downstream of the SRα promoter in pME-18Neo (Figure 1A). The expression of WT and mutant gp51 proteins by pEnv was confirmed following the transfection of COS-1 cells with either WT or mutant pEnv and western blotting analysis using anti-BLV gp51MAb (Figure 1D). No change was observed in the relative density of gp51 bands (Figure 1D lower), and the molecular masses of these mutant gp51 proteins were equivalent to those of WT (Figure 1D upper).

### 3.2. Enhanced Syncytium-Forming Ability by All Tyrosine Mutant Forms of the Infectious Molecular Clone pBLV-IF2 and Env Expression Plasmid pEnv

**Figure 2:**
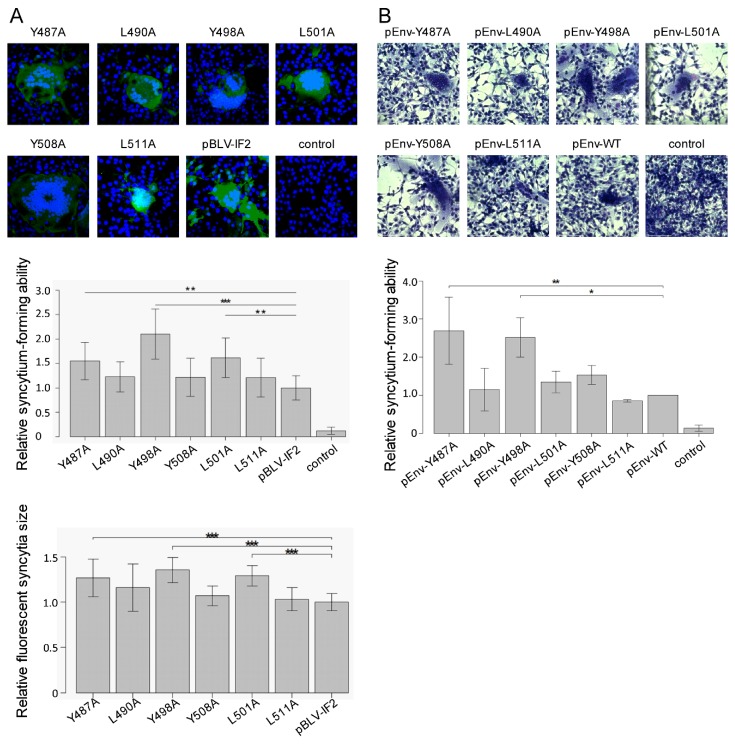
Effect on the syncytium-forming ability of mutant forms of an infectious molecular clone pBLV-IF2 and Env expression plasmid pEnv. (**A**) The luminescence syncytium induction assay (LuSIA) using CC81-GREMG cells. CC81-GREMG cells (8.0 × 10^5^) were transfected with 2.8 µg of either wild-type pBLV-IF2, mutant pBLV-IF2, or control pBluescript II SK (−) together with 1.2 µg of Flag-mRFP using 14 µg of 25 kDa linear polyethylenimine and seeded at 2 × 10^5^ cells/well in 24 well plates. At 20 h post-transfection, a fraction of cells was used to identify the ratio of RFP-expressing cells to determine the transfection efficiency. After two days post-transfection, the remaining CC81-GREMG cells were fixed, and nuclei were stained using Hoechst 33342. Fluorescent syncytia were observed using an FV-1000 fluorescence microscope (upper panel), or automatically scanned by EVOS2 fluorescence microscopy and counted computationally (middle panel). Shown are the relative numbers of fluorescent syncytia, which were normalized by the transfection efficiency and the number of cells. The fluorescence area of syncytia with EGFP was also measured using HCS Studio Cell Analysis software. Shown is the relative size of fluorescent syncytia, which were normalized by the size of pBLV-IF2 induced fluorescent syncytia for each experiment (lower panel). Each column and error bar represents the mean ± SD of fluorescent syncytia for four independent experiments. All values were analyzed by two-way ANOVA with Dunnett’s test. The asterisk indicates a statistically significant difference (* *p* < 0.05; ** *p* < 0.01, and *** *p* < 0.001). (**B**) Conventional syncytia formation assay using CC81 cells. CC81 cells (1.2 × 10^6^) were transfected with 7.2 µg of either wild-type pEnv, mutant pEnv, or control pME-18neo together with 0.8 µg of pEGFP-N1 using a mixture of 12 µL Lipofectamine 3000 Transfection Reagent and 16 µL P3000 Enhancer Regent, and seeded at 1 × 10^6^ cells in a 60 mm dish-and 2 × 10^5^ cells in a 12 well plate. At 20 h post-transfection, cells in the 12 well plate were used to identify the ratio of GFP-expressing cells to determine the transfection efficiency. After two days post-transfection, CC81 cells in the 60 mm dish were fixed and stained with May–Grunwald solution and Giemsa solution. Syncytia were counted using a microscope (upper panel). Relative numbers of syncytia normalized by the transfection efficiency are shown (lower panel). Each column and error bar represents the mean ± SD of syncytia for three independent experiments. All values were analyzed by two-way ANOVA with Dunnett’s test. The asterisk indicates a statistically significant difference (* *p* < 0.05; ** *p* < 0.01, and *** *p* < 0.001).

To determine the role of the three YXXL sequences with regard to syncytium-forming ability, we performed LuSIA using CC81-GREMG cells, which is able to visualize the syncytium formation by BLV. CC81-GREMG cells are a CC81-derived reporter cell line harboring pBLU3GREM-EGFP, which contains a mutant form of the glucocorticoid response element (GRE) in the U3 region of the BLV long terminal repeat [28], that specifically responds to BLV trans-activator p38^tax^ expression. CC81 GREMG cells were transiently transfected with infectious molecular pBLV-IF2 clones that encoded WT, Y487A, L490A, Y498A, L501A, Y508A, and L511A. Two days after transfection, syncytia expressing EGFP were observed in CC81-GREMG cells transfected with WT and mutant pBLV-IF2s (Figure 2A, upper). Notably, all three tyrosine mutations, i.e., Y487A, Y498A, and Y508A in the 1st, 2nd, and 3rd YXXL sequences, caused a significant increase in the number and size of syncytia with EGFP fluorescence as compared to that of WT pBLV-IF2. In contrast, the three leucine mutants L490A, L501A, and L511A had no effect on syncytium formation, with the numbers and size of syncytia observed being similar to those induced by WT pBLV-IF2 (Figure 2A, middle and lower).

Moreover, to evaluate the syncytium-forming ability of Env protein in the absence of other viral proteins, we performed a conventional syncytium assay using CC81 cells, which form syncytia upon BLV infection. When these cells were transfected with pEnv encoding WT, Y487A, L490A, Y498A, L501A, Y508A, or L511A, syncytia were formed in all cases (Figure 2B, right). In addition, the two tyrosine mutations Y487A and Y498A in the 1st and 2nd YXXL sequences afforded a high fusion activity in CC81 cells following transfection with an Env expression vector as determined by the numbers of syncytia. Moreover, the number of syncytia induced by Y508A in the 3rd YXXL sequence also showed a slight tendency to be increased (Figure 2B, left). In contrast, all three of the leucine mutants L490A, L501A, and L511A revealed only moderate fusion activity, as did the WT Env protein.

The results obtained by LuSIA using CC81-GREMG cells following transient transfection with WT or mutant pBLV-IF2 were similar to those observed from a conventional syncytium assay, following transient transfection with an Env expression vector into CC81 cells. Our result strongly suggested that each tyrosine residue in the 1st, 2nd, and 3rd YXXL sequence regulates the induction of syncytia by BLV.

### 3.3. Enhanced Cell Surface Expression of gp51 by All Tyrosine Mutant Forms of the Infectious Molecular Clone pBLV-IF2 and Env Expression Plasmid pEnv

**Figure 3:**
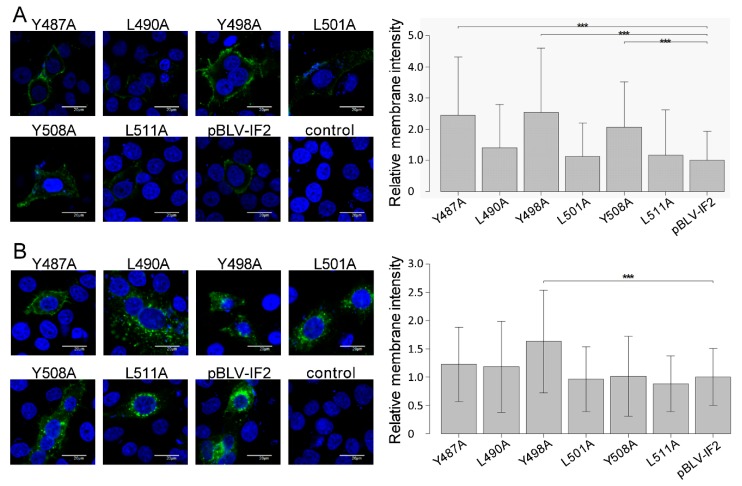
Effect on the localization of gp51 by mutant forms of the infectious molecular clone pBLV-IF2. HeLa cells (1.0 × 10^5^) were seeded on a coverslip in a 12 well plate the day prior to transfection and transfected with 2 µg of either wild-type pBLV-IF2, mutant pBLV-IF2, or control pBluescript II SK (−) using 8 μL of FuGENE HD. The transfection efficiency was similar among all mutant pBLV-IF2s as evaluated according to the ratio of GFP-expressing HeLa cells determined via FACSCalibur™ flow cytometry. (**A**) To detect cell surface gp51, cells were fixed and stained with anti-gp51 MAb, followed by Alexa Fluor 488-conjugated anti-Mouse IgG, then stained with Hoechst 33342 and observed using an FV-1000 fluorescence microscope (right panel). (**B**) To detect intracellular Env protein, cells were fixed, permeabilized with 0.5% Triton X-100, stained with anti-BLVgp51 MAb followed by Alexa Fluor 488-conjugated anti-Mouse IgG, and observed using an FV-1000 fluorescence microscope (right panel). (**A**,**B**) Fluorescence intensity maps were plotted for linear transects drawn through the nuclei by line scan measurements through each cell using FV10-ASW 4.02 microscope software, and fluorescence intensities on the cell surface were measured. The width of each line was thinner than 1 pixel. Peak membrane intensity was normalized by the mean intensity of pBLV-IF2 for each experiment. The results show the relative intensities of at least 50 cells expressing gp51 over seven independent experiments. Each column and error bar represents the mean ± SD of intensity for all cells. All values were analyzed by two-way ANOVA with Dunnett’s test. The asterisk indicates a statistically significant difference (* *p* < 0.05; ** *p* < 0.01, *** *p* < 0.001).

In the case of HIV, cell surface-associating Env protein can trigger cell–cell fusion [35]. Therefore, to gain insight into the mechanisms of the enhanced fusion activity of gp51 protein by each of the three tyrosine mutations in the three YXXL sequences, surface expression of the gp51 protein on HeLa cells following transient transfection with WT or mutant pBLV-IF2 was evaluated using indirect immunofluorescence analysis without TritonX-100 permeabilization, and the intensity differences of the two signal of cells analyzed by line scan measurements through each cell using FV10-ASW 4.02 microscopy software. Notably, all tyrosine mutations caused a drastic enhancement of fluorescence on the cell surface (Figure 3A, right). In contrast, HeLa cells transfected with any of the three leucine mutant pBLV-IF2s, such as L490A, L501A, and L511A, revealed slight fluorescence at the cell membrane as did cells transfected with WT pBLV-IF2. When fluorescence intensities on the cell surface were measured using FV10-ASW 4.02 microscope software, the intensity of gp51 detected at the cell surface in HeLa cells transfected with pBLV-IF2s encoding the three tyrosine mutants was significantly greater than that of HeLa cells transfected with pBLV-IF2 encoding WT or the three leucine mutants (Figure 3A, left).

To confirm the distribution of gp51 protein in the cytoplasm, gp51 protein was detected under the condition of permeabilization by TritonX-100. In the cytoplasm of HeLa cells transfected with pBLV-IF2s that encoded WT or the three leucine mutants L490A, L501A, or L511A, or one of two tyrosine mutants, Y487A or Y508A, gp51 protein was predominantly localized in punctate cytoplasmic structures around the nucleus, with lesser amounts in the cell surface membrane (Figure 3B, right). Notably, gp51 protein was predominantly localized at the surface of the cellular membrane and slightly in punctate cytoplasmic structures around the nucleus in HeLa cells transfected with pBLV-IF2 that encoded only Y498A. Similarly, the intensity of gp51 detected at the cell surface in HeLa cells that were transfected with pBLV-IF2s that encoded only Y498A was significantly greater than that of HeLa cells transfected with other pBLV-IF2s (Figure 3B, left). These results suggested that each tyrosine of the YXXL sequence mediated Env localization and that the altered Env localization from the cytoplasm to the membrane consequent to tyrosine to alanine mutation-induced greater syncytium-forming ability. In addition, it was shown that Triton X-100 treatment led to the release of a portion of membrane proteins from the cell membrane [36]. Here we found that Y498A Env exhibits a higher membrane localization rate under the permeabilized condition compared to that of WT Env, suggesting that Y498A Env might be transported to the detergent-resistant membrane, thereby leading to the reduction of internalization.

**Figure 4:**
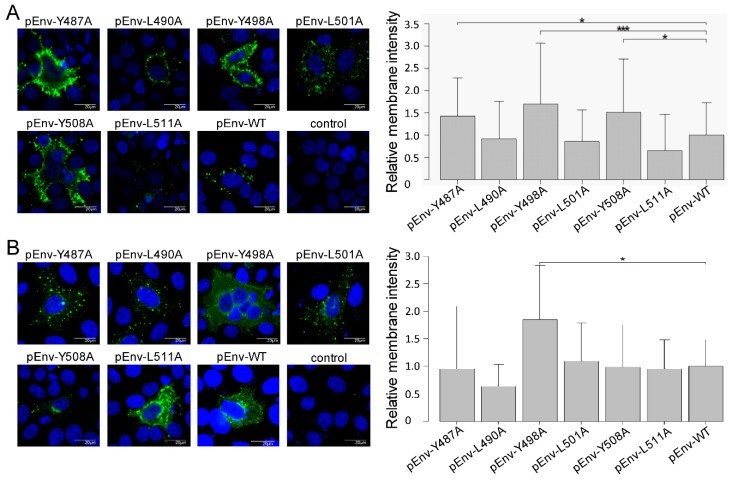
Effect on the localization of gp51 by mutant forms of the Env expression plasmid pEnv. HeLa cells (1.0 × 10^5^) were seeded on a coverslip in a 12 well plate the day prior to transfection and transfected with 2 µg of either wild-type pEnv, mutant pEnv, or the control pME-18neo using 8 μL of FuGENE HD. (**A**) To detect cell surface gp51, cells were fixed and stained using an anti-gp51 MAb, followed by Alexa Fluor 488-conjugated anti-mouse IgG. (**B**) To detect intracellular gp51, cells were fixed, permeabilized with 0.5% Triton X-100, and stained with an anti-gp51 MAb followed by Alexa Fluor 488-conjugated anti-mouse IgG. (**A**,**B**) Fluorescence intensity maps were plotted for linear transects drawn through the nuclei by line scan measurements through each cell using FV10-ASW 4.02 microscope software, and fluorescence intensities on the cell surface were measured. The width of each line was thinner than 1 pixel. Peak membrane intensity was normalized by the mean intensity of pEnv for each experiment. The results show the relative intensities of at least 50 cells expressing gp51 over six independent experiments. Each column and error bar represents the mean ± SD of intensity for all cells. All values were analyzed by two-way ANOVA with Dunnett’s test. The asterisk indicates a statistically significant difference (* *p* < 0.05; ** *p* < 0.01, and *** *p* < 0.001).

Furthermore, the results using an infectious molecular clone were confirmed by localization of Env protein without other viral proteins using an Env expression vector, as shown in Figure 4A,B. This result suggested that each tyrosine of the three YXXL sequences mediated Env localization without other viral proteins.

To further demonstrate membrane domain-specific localizations of gp51, we performed Z-stack analysis from three images per cell among a total of 10 HeLa cells transiently transfected with WT and all mutant forms of the infectious molecular clone pBLV-IF2, as well as Env expression plasmid pEnv. A presentative of all transfectants can be seen in Supplementary Figures S2 and S3. We confirmed the enhanced cell surface expression of tyrosine mutated gp51 in both pBLV-IF2 (Figure 3) and pEnv (Figure 4) transfected cells by Z-stack analysis.

### 3.4. Localization of gp51 Decreases at the Early Endosome Despite no Effects at the Trans-Golgi Network by Tyrosine Mutant Forms of the Infectious Molecular Clone pBLV-IF2

**Figure 5:**
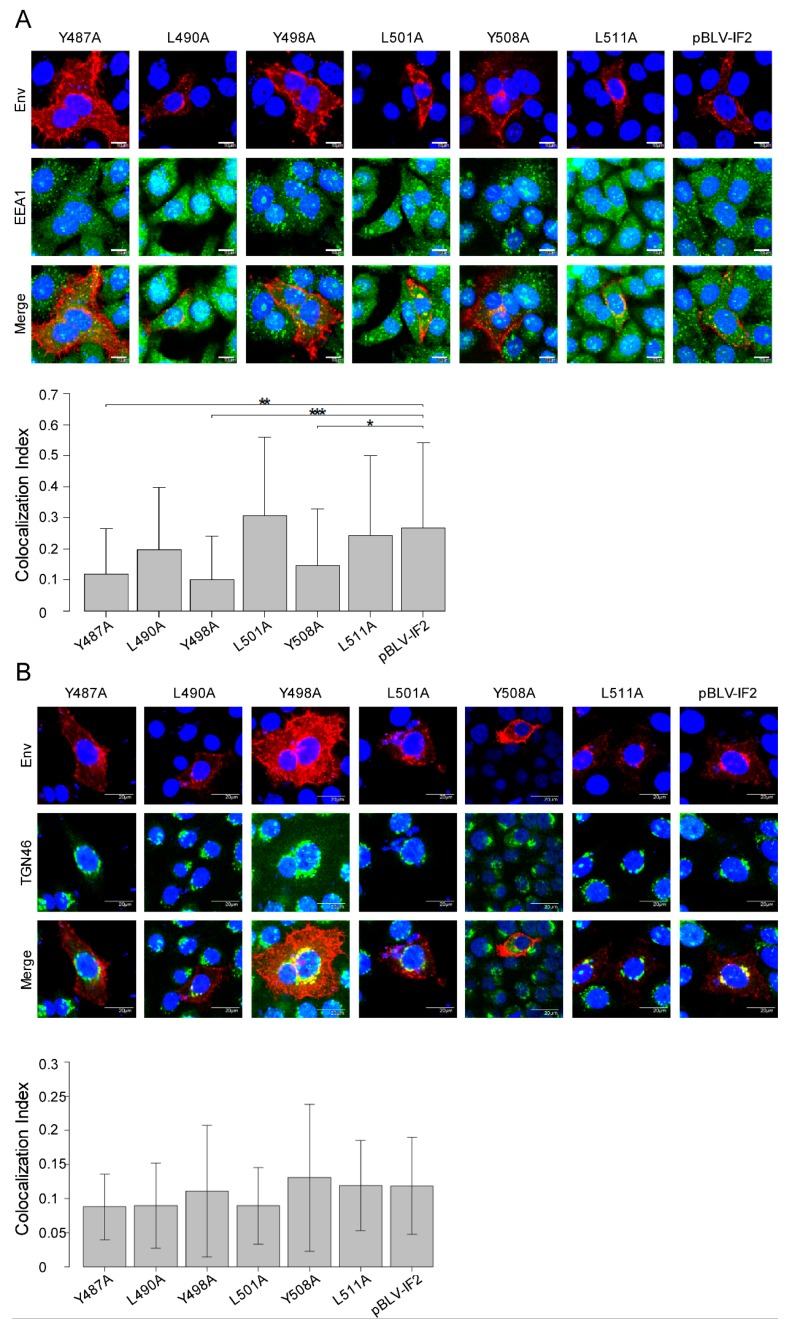
Localization of gp51 in the early endosome and trans-Golgi network. HeLa cells (1.0 × 10^5^) were seeded on a coverslip in a 12 well plate the day prior to transfection and transfected with 2 µg of either wild-type pBLV-IF2, mutant pBLV-IF2, or control pBluescript II SK (−) using 8 μL of FuGENE HD. (**A**) To detect the localization of gp51 in the early endosome, cells were fixed, permeabilized with 0.5% Triton X-100, and stained with anti-BLV gp51 MAb followed by Alexa Fluor 594-conjugated anti-mouse IgG and anti-EEA1 polyclonal antibody (PAb), followed by Alexa Fluor 488-conjugated goat anti-rabbit IgG (upper panel). The colocalization index between gp51 and EEA1 was calculated using Villalta’s algorithm with FV10-ASW 4.02 microscope software (lower panel). The results show the relative colocalization index between gp51 and EEA1 of at least 50 cells over seven independent experiments. Each column and error bar represents the mean ± SD of intensity for all cells. All values were analyzed by two-way ANOVA with Dunnett’s test. (**B**) To detect the localization of gp51 in the trans-Golgi network, cells were fixed, permeabilized with 0.5% Triton X-100, and stained with anti-BLV gp51 MAb followed by Alexa Fluor 594-conjugated goat anti-mouse, and anti-TGN46 PAb followed by Alexa Fluor 488-conjugated goat anti-rabbit IgG (upper panel). The colocalization index between gp51 and TGN46 was calculated using Villalta’s algorithm on FV10-ASW 4.02 microscope software (Lower panel). The results show the relative colocalization index between gp51 and TGN46 of at least 50 cells over three independent experiments. Each column and error bar represents the mean ± SD of intensity for all cells. All values were analyzed by two-way ANOVA with Dunnett’s test. The asterisk indicates a statistically significant difference (*p < 0.05; **p < 0.01, and ***p < 0.001).

The location of Env protein as punctate cytoplasmic structures around the nucleus in the cytoplasm implies that it might be clustered within an organelle. Protein transport is strongly involved in the localization of proteins in organelles, such as the Golgi apparatus, endosomes, and lysosomes [37]. Therefore, to clarify the role of the YXXL sequence in the transport of Env, it is important to reveal the organelle, such as the trans-Golgi network or early, late, or recycling endosome, in which the gp51 protein is located. Accordingly, to investigate whether Env protein localized in the early endosome, we visualized the subcellular localization of gp51 and the early endosome marker EEA1 [38] in HeLa cells, following transient transfection with WT or mutant pBLV-IF2 using confocal laser scanning microscopy (Figure 5A). Cells were labeled with an anti-BLV gp51 MAb (red fluorescence) to detect the Env protein and an anti-EEA1 (green fluorescence) antibody to detect EEA1, and stained with Hoechst 33342 (blue fluorescence) for detection of the nucleus (Figure 5A, upper). The colocalization index between gp51 and EEA1 was calculated using FV10-ASW 4.02 microscope software (Figure 5A, lower). In HeLa cells transfected with pBLV-IF2s encoding WT protein or the three leucine mutants L490A, L501A, or L511A, the colocalization signal (yellow) of EEA1 (green) and gp51 (red) mainly coexisted in the early endosome. However, all tyrosine mutations caused a significant decrease of colocalization between gp51 and EEA1 in the early endosome (Figure 5A, lower) because gp51 mainly localized at the cell surface. Next, to investigate whether Env protein localizes in the trans-Golgi network, we visualized the distribution of gp51 and the trans-Golgi network marker TGN46 [39]. As monitored by immunostaining for anti-BLV gp51 MAb (red fluorescence) and anti-TGN46 (green fluorescence) antibodies, approximately 10% (yellow) of gp51 (red) colocalized with TGN46 (green), in all of HeLa cells transiently transfected with WT or mutant pBLV-IF2s, and the amounts of colocalization (yellow) between gp51 and TGN46 in the trans-Golgi network were not affected by any of the YXXL mutations (Figure 5B). Our results clearly suggested that Env proteins on HeLa cells transfected with pBLV-IF2s encoding WT or the three leucine mutants were synthesized in the rough-surfaced endoplasmic reticulum and transported to the cell membrane via the trans-Golgi network, and then internalized into the early endosome by endocytosis. However, for all of the tyrosine mutants Y487A, Y498A, and Y508A, although a portion of the Env protein is able to internalize into the endosome, a large fraction is not internalized therein, instead, remaining and enhancing the levels on the cell membrane. In particular, Env protein produced by Y498A appears to be transported to the detergent-resistant membrane, which might constitute one of the reasons why internalization is interrupted.

### 3.5. Y498A and L511A Mutants Interrupt the Incorporation of gp51 into Virions

**Figure 6:**
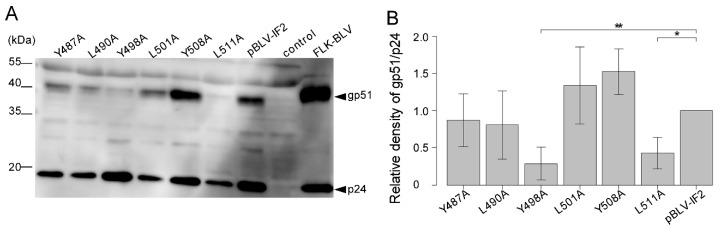
Effect of incorporation of gp51 into the virion by mutant forms of the infectious molecular clone pBLV-IF2. COS-1 cells (5.0 × 10^5^) were seeded in a 60 mm dish the day prior to transfection and transfected with 7.6 µg of either wild-type pBLV-IF2, mutant pBLV-IF2, or the control Bluescript II SK (−) vector together with 0.4 µg of pEGFP-N1 using 32 μL of FuGENE HD. (**A**) Virus particles were collected from the supernatants of the cells and subjected to western blotting analysis with anti-BLV gp51 MAb and anti-BLV p24 MAb followed by horseradish peroxidase-conjugated goat anti-mouse IgG. Positions of the molecular mass marker and the BLV structural protein are indicated. (**B**) For quantification, densities of bands were analyzed using ImageJ software. Densities of gp51 were normalized with those of p24. Each column and error bar represents the mean ± SD of density for six independent experiments. All values were analyzed by two-way ANOVA with Dunnett’s test. The asterisk indicates a statistically significant difference (* *p* < 0.05; ** *p* < 0.01).

The intracellular distribution of HIV-1 Env protein plays an important role in HIV-1 assembly [21]. Our results also showed that all tyrosine mutations in the three YXXL sequences each altered the localization of the Env protein from the cell surface to the cytoplasm. Therefore, we examined the effect of the three YXXL sequences on the incorporation of the Env protein into virions. As shown in Figure 1A, the bands of cell-associated viral proteins gp51, gp30, and p24 were similar for each of the six mutant proviruses and the WT provirus. Next, we analyzed concentrated virus particles that had been released from COS-1 cells that were transiently transfected with each of the six mutant and WT proviruses by western blotting analysis using anti-gp51 and anti-p24 MAbs (Figure 6A). The relative density of bands corresponding to structural protein p24 was similarly detected in virion produced by each of the six mutant proviruses and the WT provirus (Figure 6B). However, no bands of the gp51 protein were observed in virions produced by Y498A and L511A in the 2nd and 3rd YXXL sequence mutants, respectively. This result suggested that the incorporation of Env protein into virions is controlled by the 2nd and 3rd YXXL sequences.

### 3.6. Effect of Mutations in the YXXL Sequences on Syncytium-Forming Ability, gp51 Localization, and gp51 Incorporation into Virions

As summarized in Table 1, our results indicated that all tyrosine mutations enhanced the syncytium-forming ability and altered the localization of gp51 from the cytoplasm to the cell membrane. In addition, the amounts of Env protein induced by all tyrosine mutants in the early endosome were slightly decreased. However, only Y498A among the tyrosine mutants and L511A among the leucine mutants interrupted the incorporation of Env protein into the virion. Thus, our findings strongly suggested that the three YXXL sequences independently regulate the viral life cycle.

## 4. Discussion

**Figure 7:**
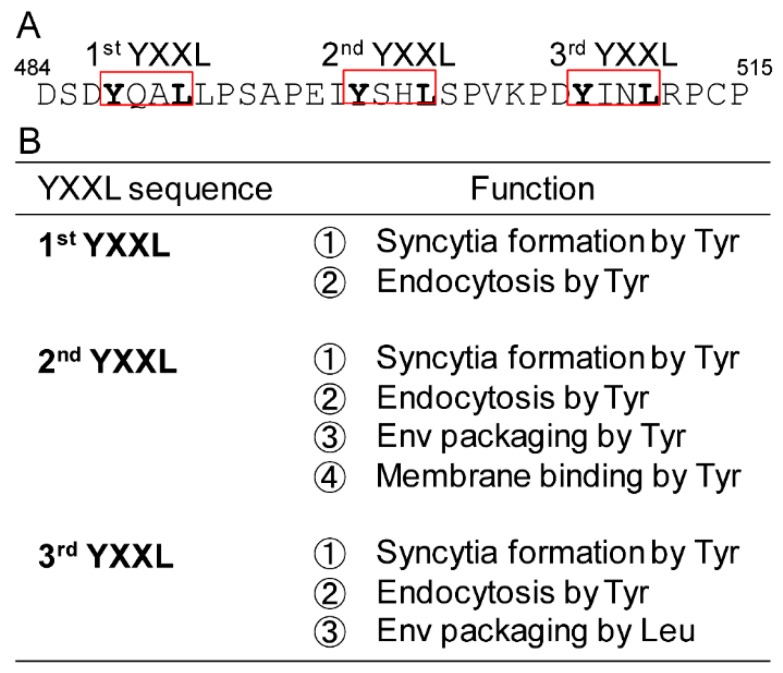
Schematic representations of YXXL sequences and the new function discovered in this study. (**A**) All three YXXL sequences are completely conserved among BLV variants. The YXXL sequences were named as 1st YXXL, 2nd YXXL, and 3rd YXXL. (**B**) The multiple functions of each YXXL sequence were revealed in this study.

Here, we examined the role of the three YXXL sequences in BLV gp30 in the viral life cycle, focusing on the syncytium-forming ability, Env protein distribution, and incorporation of Env protein into the virion, using an infectious molecular clone and Env expression vector (Table 1). We identified novel functions of each YXXL sequence (Figure 7), reaching three major conclusions. First, the present study revealed that each tyrosine residue in all three YXXL sequences independently regulates syncytia forming ability by the Env protein. Notably, this newly identified activity of the YXXL sequence was the most likely to depend upon the control of the amount of Env protein at the cell membrane by the relevant tyrosine residue. This is the first report showing an association between syncytium-forming ability and the three YXXL sequences of gp30. Second, our results also demonstrated that each tyrosine residue in the three YXXL sequences differently affected the localization of Env proteins at the cell surface in both the presence and absence of other viral proteins, as determined using an infectious molecular clone and the Env expression vector, respectively. For example, the results of colocalization between the Env protein and EEA1 or TGN46 in the cytoplasm suggested that the YXXL sequence mediates internalization of the Env protein following its transport to the cell surface. In addition, we revealed that only the Env protein produced by the Y498A mutation in the 2nd YXXL sequence was located at the cell membrane under the permeabilized condition, indicating that the tyrosine residue in the 2nd YXXL sequence mediates the localization of Env protein in membrane domain, which might also be associated with incorporation of Env protein. Third, our data showed that tyrosine in the 2nd YXXL sequence and leucine in the 3rd YXXL sequence regulates the incorporation of the Env protein into virions by functionally distinct mechanisms, with likely interactions between Env protein incorporation and internalization. Thus, each of the three YXXL sequences appeared to play critical roles in the viral life cycle independently.

As summarized in Figure 7, the 1st YXXL was required for syncytium-forming ability under the control of the amount of Env protein at the cell membrane mediated by the tyrosine residue. The 2nd YXXL regulates not only syncytium forming ability but also the incorporation of the Env protein into viral particles as mediated by the tyrosine residue. In addition, the 3rd YXXL sequence regulates syncytium forming ability by the tyrosine residue in addition to the incorporation of the Env protein into viral particles by the leucine residue. Our findings suggested that each YXXL sequence may independently regulate the Env protein via individual host factors. Notably, this represents a distinguishing feature between the YXXL sequences of BLV and those of other retroviruses, as the Env protein of most retroviruses such as HIV, SIV, and HTLV-1 contains only a single YXXφ motif, whereas BLV possesses three YXXL sequences, each of which is independently involved in an essential role in the viral life cycle. This appears to be the reason why the three YXXL sequences are completely conserved among the nucleotide sequences of 517 BLV strains collected from GenBank (data not shown). In addition, our findings suggest that these completely conserved sequences also contribute to the ability of BLV to escape Env protein-specific host immune responses by limiting the amount of Env protein on the cells to the minimum that is necessary for effective virus production.

Retroviral Env protein has a central role in virus infectivity and syncytia formation [40]. In the case of BLV, several single amino acid substitutions in gp30 downregulated syncytia formation [41,42]. In contrast, our results demonstrated that alanine mutation at the tyrosine residue in the three YXXL sequences in gp30 of BLV independently caused a marked enhancement of syncytia-forming ability by the Env protein. Thus, it appears likely that modification of the retroviral TM protein involving a deletion or site-directed mutation affects syncytium-forming ability by two plausible mechanisms. First, modification of the TM protein directly affects fusion activity without changing the expression level of Env protein on the cell surface. For example, a 146 amino acid C-terminal truncation of the cytoplasmic domain of the TM protein of macaque SIV altered the conformation of its external domain and enhanced syncytium formation [43]. Second, modification of the TM protein affects fusion activity consequent to modulation of the expression level of the Env protein on the cell surface. A single mutation of tyrosine to cysteine at position 723 of the SIVmac239 TM protein increased the level of Env protein on the cell surface and significantly increased the fusion activity [44]. Here, the marked enhancement of syncytium-forming ability induced by tyrosine mutation of the BLV YXXL sequences was likely dependent on the latter mechanism as the Env protein induced by all tyrosine mutants increased the level of expression on the cell surface.

Retroviral Env protein trafficking to the assembly site on the cell membrane is regulated by several different mechanisms. For example, gp41, which is the Env protein transmembrane subunit of HIV-1, interacts with AP-1 to facilitate traffic to the membrane, AP-2 for endocytosis, and Rab11-FIP1C for recycling to the membrane [18,45,46]. However, the trafficking pathway to the budding site and the trafficking host factors of BLV gp51 and gp30 remain unclear. In the present study, the amounts of Env protein at the cell surface produced by all tyrosine mutants significantly increased under conditions with other viral proteins as shown using an infectious molecular clone. This result was confirmed by localization of the Env protein without other viral proteins, as mediated using an Env expression vector. Moreover, a tendency for decreased colocalization levels of EEA1 and Env protein in the early endosome was observed in tyrosine mutants as compared with that of the WT, indicating that the tyrosine residue in the YXXL sequence may mediate endocytosis of the Env protein. In contrast, no change was observed in the colocalization level of TGN46 and gp51 among all mutant and WT Env proteins, indicating that all tyrosine mutants are synthesized in the rough-surfaced endoplasmic reticulum and trafficked to the cell membrane via the trans-Golgi network at the same levels as those of WT gp51 proteins, whereupon WT gp51 proteins are immediately internalized in early endosomes whereas all tyrosine mutants are retained at the membrane. This role in Env endocytosis was supported by several previous findings. Specifically, several studies using a gp30 cytoplasmic tail and CD8 chimera protein (CD8-CTM) have also reported that gp30 mediates endocytosis [23]. In particular, Y487A and Y498A mutations in the 1st and 2nd YXXL sequences increase the levels of CD8-CTM on the cell surface compared with that of WT [22]. Taken together with these previous results, our findings suggest that Env protein is trafficked to the membrane as follows: (i) Env proteins are synthesized in the rough-surfaced endoplasmic reticulum as directed by the Env protein signal peptide. (ii) Env proteins are matured and transported to the cell membrane via the trans-Golgi network. (iii) Env proteins on the cell membrane are immediately internalized into the endosome by endocytosis. Collectively, we suggest that the three YXXL sequences separately play an important role in trafficking the BLV Env protein to the cell membrane assembly site, as described above. In addition, the colocalization index between gp51 and EEA1 in HeLa cells transfected with pBLV-IF2 was slightly higher than that between gp51 and TGN46. This result also suggested that Env proteins on the cell membrane are immediately internalized to escape Env protein-specific host immune responses.

Translocation of retroviral Env proteins to the budding site is essential to their incorporation into virions, and viral replication kinetics appears to be associated with Env incorporation [21]. In the case of HIV, Env protein is trafficked to the bidding site through the endosomal recycling compartment, with this being required for particle incorporation [47]. In our study, only the Y498A mutation among the three tyrosine mutations of YXXL sequences interrupted the incorporation of Env protein into virions. This interruption by Y498A has already been observed in a previous study [15], although the mechanism remains unclear. In the present study, only the Env protein produced by the Y498A mutation in the 2nd YXXL sequence was located at the cell membrane under permeabilized conditions. Therefore, incorporation into viral particles may be inhibited by not only the change of Env trafficking but also the change of Env protein localization in the membrane domain. Similarly, the L511A mutation in the 3rd YXXL sequence interrupted the incorporation of Env protein into virions. However, the Env protein produced by the L511A mutation might exhibit interrupted Gag-Env interaction or other mechanisms of Env escort to the budding site because L511A did not affect Env localization. Further study is required to define the detail mechanisms underlying the incorporation into viral particles and clarify the novel functions of YXXL sequences.

Our results strongly indicated that mediation of syncytia formation by YXXL sequences appears to depend mainly on the regulation of Env traffic. In particular, it is considered that tyrosine mutations inhibit the binding with AP2-µ2 and interrupt endocytosis of the Env protein after transport to the cell membrane because YXXL sequences fit the AP2-µ2 binding motif. Although an interaction between gp30 and AP2-µ2 has been suggested, direct binding of gp30 and AP2-µ2 has not yet been reported [23]. Further analysis of the direct binding of gp30 and AP2-µ2 is required to clarify Env protein traffic. However, change in the Env protein traffic is not completely able to explain why only Y498A among tyrosine and only L511A among leucine mutants could inhibit the incorporation of Env protein into virions, suggesting the existence of other host factors that interact with the YXXL sequence and regulate the viral life cycle. Therefore, identification of host factors that directly or indirectly interact with YXXL sequences may lead to an improved understanding of the viral life cycle, and would serve as the first step in the development of novel drugs against BLV.

## Figures and Tables

**Table 1:** Effect on the localization of the Env protein, syncytium-forming ability, and Env protein incorporation into virions of the substitution of YXXL sequences in the infectious molecular clones or Env expression vectors.

Plasmid	YXXL Sequence	Mutation	Syncytium-Forming Ability ^1^	Localization			Incorporation in Virions ^5^
				Whole ^2^	Early Endosome ^3^	Trans-Golgi Network ^4^	
pBLV-IF2	1st YXXL	Y487A	+++	M	−	+	+
		L490A	+	C	+	+	+
	2nd YXXL	Y498A	++++	M	−	+	−
		L501A	+	C	+	+	+
	3rd YXXL	Y508A	+++	M	−	+	+
		L511A	+	C	+	+	−
		WT	+	C	+	+	+
pEnv	1st YXXL	Y487A	++	M			
		L490A	+	C			
	2nd YXXL	Y498A	++	M			
		L501A	+	C			
	3rd YXXL	Y508A	+	M			
		L511A	+	C			
		WT	+	C			

^1^ Significance level of altered syncytium-forming ability compared with that of wild-type: +, wild-type level; ++, *p* < 0.05; +++, *p* < 0.01; ++++, and *p* < 0.001. ^2^ M and C indicate that the Env protein mainly localized in the membrane and cytoplasm, respectively. ^3^ Localization rate of the Env protein in the early endosome: −, below wild-type; +, wild-type level. ^4^ Localization rate of the Env protein in the trans-Golgi network: +, wild-type level. ^5^ Level of incorporation of the Env protein into virions: −, below wild-type; +, wild-type level.

## Supplementary Materials

The following are available online at https://www.mdpi.com/1999-4915/11/12/1140/s1, Figure S1: Quantification of intensity of Env protein on the cell membrane by line scan measurement, Figure S2: Z-stack analysis of localization of gp51 by mutant forms of the infectious molecular clone pBLV-IF2 in three optical section, Figure S3: Z-stack analysis of localization of gp51 by mutant forms of the Env expression plasmid pEnv in three optical section.
