# Supplementary material for: Three YXXL Sequences of a Bovine Leukemia Virus Transmembrane Protein are Independently Required for Fusion Activity by Controlling Expression on the Cell Membrane
Source: Viruses. 2019 Dec 10;11(12):1140. doi: 10.3390/v11121140 (PMC6950344; doi:10.3390/v11121140)
Supplement: Supplementary file 1 [file viruses-11-01140-s001.pdf]

viruses

MDPI

Supplemental figure

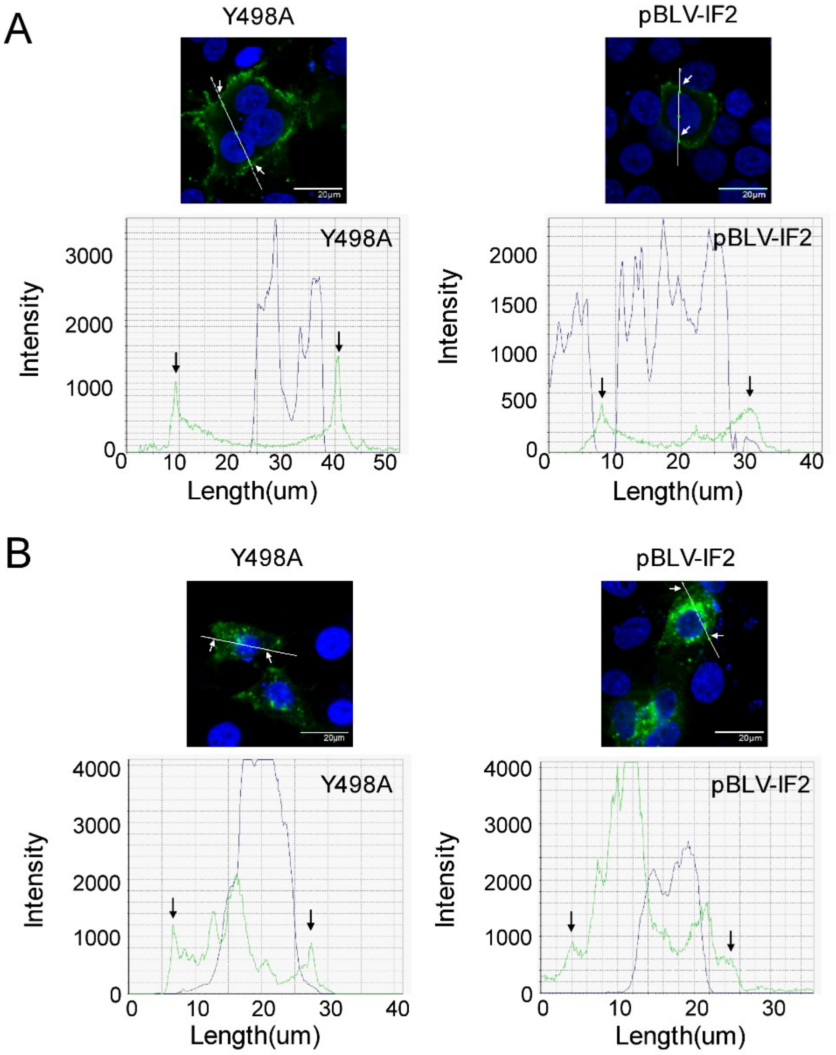

Figure S1. Quantification of intensity of Env protein on the cell membrane by line scan measurement. HeLa cells (1.0 × 10^5) were seeded on a coverslip in a 12 well plate the day prior to transfection and transfected with each pBLV-IF2, each pEnv, or control vector using FuGENE HD. (**A**) To detect cell surface gp51, cells were fixed and stained using an anti-gp51 MAb followed by Alexa Fluor 488-conjugated anti-Mouse IgG. (**B**) To detect intracellular gp51, cells were fixed, permeabilized with 0.5% Triton X-100, and stained with an anti-gp51 MAb followed by Alexa Fluor 488-conjugated anti-Mouse IgG. (**A** and **B**) Fluorescence intensity maps were plotted for linear transects drawn through the nuclei by line scan measurements using FV10-ASW 4.02 microscope software and fluorescence intensities on the cell surface were measured from these data (lower panel). Width of each line were thinner than 1 pixel. White in photo and black in graph arrows indicate that positions of the plasma membrane.

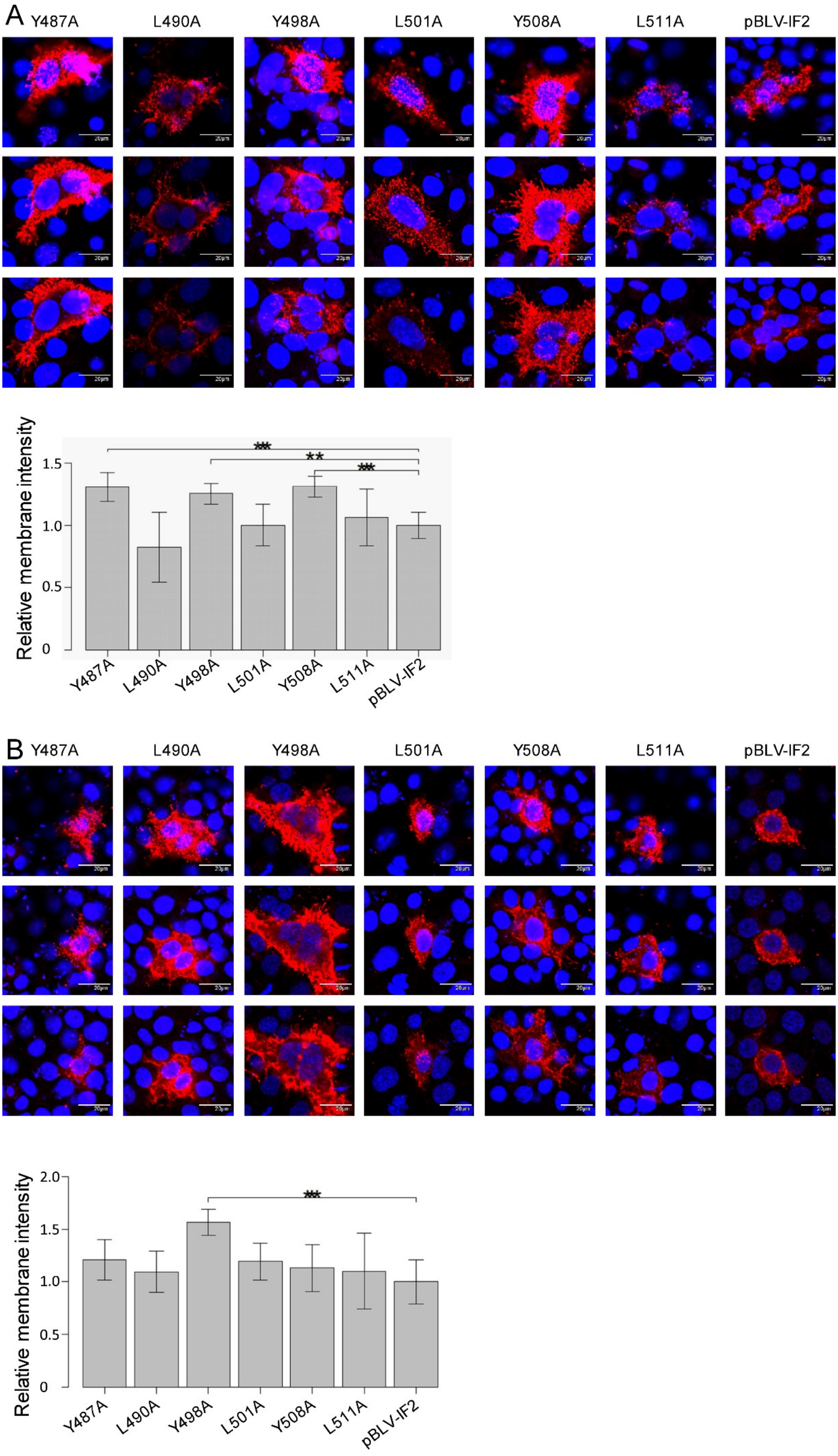
A
Y487A
L490A
Y498A
L501A
Y508A
L511A
pBLV-IF2
Relative membrane intensity
0
0.5
1.0
1.5
***
**
***
B
Y487A
L490A
Y498A
L501A
Y508A
L511A
pBLV-IF2
Relative membrane intensity
0
0.5
1.0
1.5
2.0
***

Figure S2. Z-stack analysis of localization of gp51 by mutant forms of the infectious molecular clone pBLV-IF2 in three optical section. HeLa cells (1.0×10^5) were seeded on a coverslip in a 12 well plate the day prior to transfection and transfected with 2 μg of either wild-type pBLV-IF2, mutant pBLV-IF2, or control pBluescript II SK (−) using 8 μL of FuGENE HD. (**A**) To detect cell surface gp51, cells were fixed and stained using an anti-gp51 MAb followed by Alexa Fluor 594-conjugated anti-Mouse IgG (upper panel). (**B**) To detect intracellular Env protein, cells were fixed, permeabilized with 0.5% Triton X-100, stained with anti-BLVgp51 MAb followed by Alexa Fluor 594-conjugated anti-Mouse IgG, and observed using an FV-1000 fluorescence microscope (upper panel). (**A** and **B**) For Z-stack analysis, three images were recorded at 2.0 μm intervals. Fluorescence intensities on the cell surface were measured by line scan measurements through each cell using FV10-ASW 4.02 microscope software (under panel) and normalized by the mean intensity of pBLV-IF2. The results show the relative intensities of 10 cells expressing gp51. Each column and error bar represents the mean ± SD of intensity for all cells. All values were analyzed by two-way analysis of variance (ANOVA) with the Dunnett test. The asterisk indicates a statistically significant difference ($^*p < 0.05$; $^{**}p < 0.01$, $^{***}p < 0.001$).

A

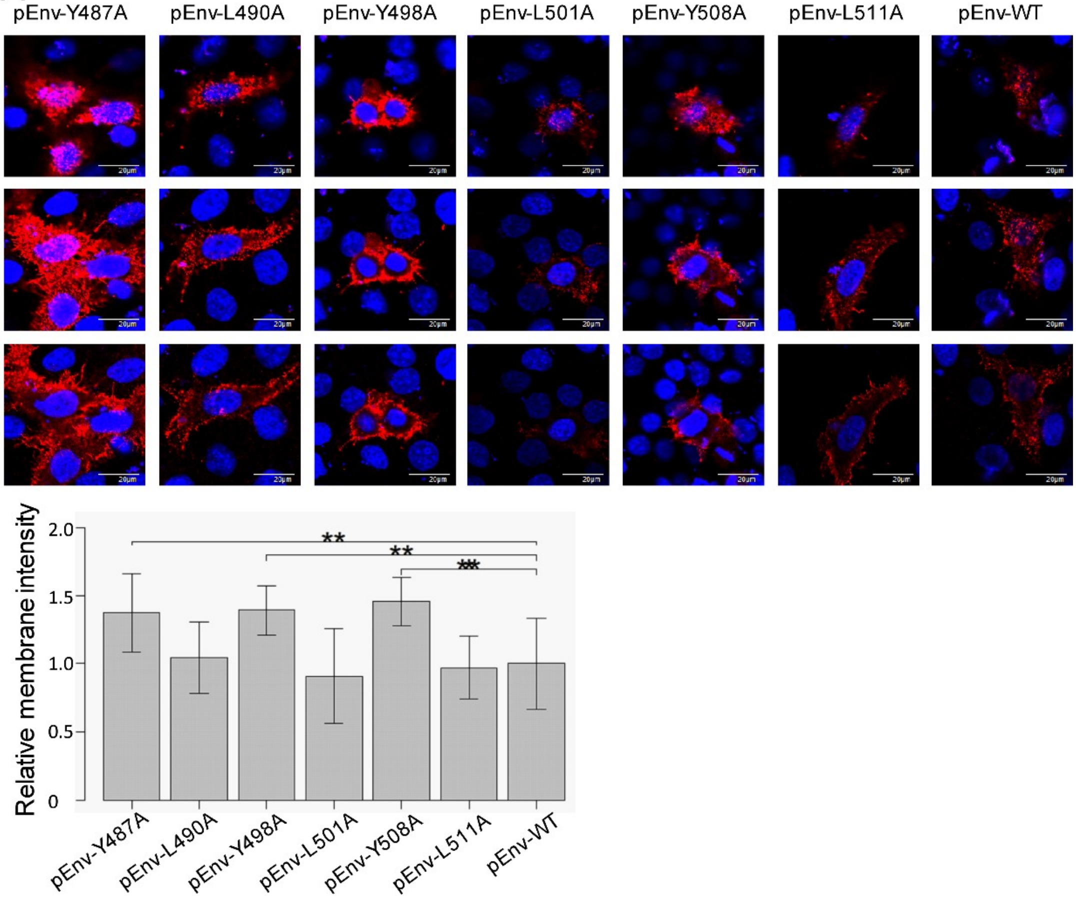
pEnv-Y487A
pEnv-L490A
pEnv-Y498A
pEnv-L501A
pEnv-Y508A
pEnv-L511A
pEnv-WT
Relative membrane intensity
2.0
1.5
1.0
0.5
0
**
**
***

B

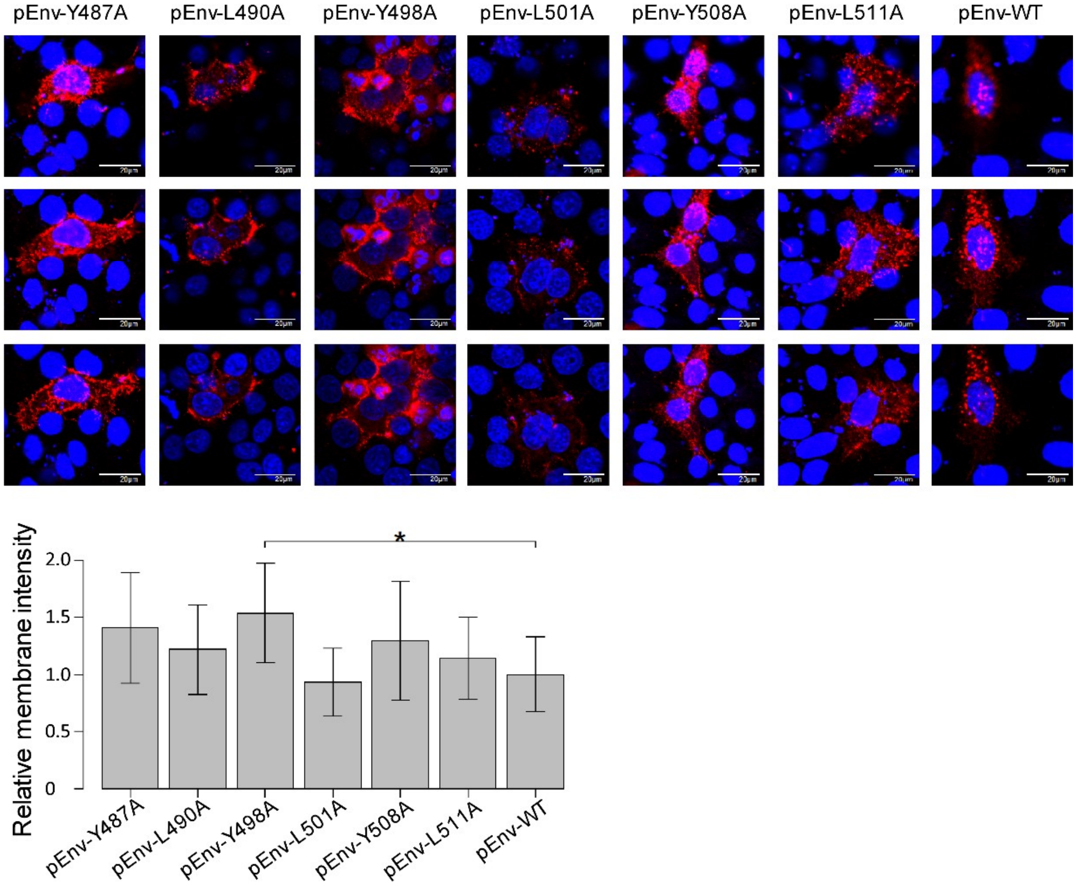
pEnv-Y487A
pEnv-L490A
pEnv-Y498A
pEnv-L501A
pEnv-Y508A
pEnv-L511A
pEnv-WT
Relative membrane intensity
2.0
1.5
1.0
0.5
0
*

Figure S3. Z-stack analysis of localization of gp51 by mutant forms of the Env expression plasmid pEnv in three optical section. HeLa cells (1.0×10^5) were seeded on a coverslip in a 12 well plate the day prior to transfection and transfected with 2 μg of either wild-type pEnv, mutant pEnv, or the control pME-18neo using 8 μL of FuGENE HD. (**A**) To detect cell surface gp51, cells were fixed and stained using an anti-gp51 MAb followed by Alexa Fluor 594-conjugated anti-Mouse IgG (upper panel). (**B**) To detect intracellular Env protein, cells were fixed, permeabilized with 0.5% Triton X-100, stained with anti-BLVgp51 MAb followed by Alexa Fluor 594-conjugated anti-Mouse IgG, and observed using an FV-1000 fluorescence microscope (upper panel). (**A** and **B**) For Z-stack analysis, three images were recorded at 2.0 μm intervals. Fluorescence intensities on the cell surface were measured by line scan measurements through each cell using FV10-ASW 4.02 microscope software (under panel) and normalized by the mean intensity of pEnv-WT. The results show the relative intensities of 10 cells expressing gp51. Each column and error bar represents the mean ± SD of intensity for all cells. All values were analyzed by two-way analysis of variance (ANOVA) with the Dunnett test. The asterisk indicates a statistically significant difference (*$p < 0.05$; **$p < 0.01$, ***$p < 0.001$).
